# Specific feature recognition on group specific networks (SFR-GSN): a biomarker identification model for cancer stages

**DOI:** 10.3389/fgene.2024.1407072

**Published:** 2024-05-23

**Authors:** Bolin Chen, Yuxin Wang, Jinlei Zhang, Yourui Han, Hamza Benhammouda, Jun Bian, Ruiming Kang, Xuequn Shang

**Affiliations:** ^1^ School of Computer Science, Northwestern Polytechnical University, Xi’an, Shaanxi, China; ^2^ Key Laboratory of Big Data Storage and Management, Northwestern Polytechnical University, Ministry of Industry and Information Technology, Xi’an, Shaanxi, China; ^3^ Department of General Surgery, Xi’an Children’s Hosptial, Xi’an Jiaotong University Affiliated Children’s Hosptial, Xi’an, China; ^4^ Rewise (Hangzhou) Information Technology Co., Ltd, Hangzhou, China

**Keywords:** biomarker, cancer stages, group specific network, multi classification tasks, edge feature

## Abstract

**Background and Objective:**

Accurate identification of cancer stages is challenging due to the complexity and heterogeneity of the disease. Current clinical diagnosis methods primarily rely on phenotypic observations, which may not capture early molecular-level changes accurately.

**Methods:**

In this study, a novel biomarker recognition method was proposed tailored for cancer stages by considering the change of gene expression relationships. Utilizing the sample-specific information and protein-protein interaction networks, the group specific networks were constructed to address the limited specificity of potential biomarkers. Then, a specific feature recognition method was proposed based on these group specific networks, which employed the random forest algorithm for initial screening followed by a recursive feature elimination process to identify the optimal biomarker subset. During exploring optimal results, a strategy termed the Cost-Benefit Ratio, was devised to facilitate the identification of stage-specific biomarkers.

**Results:**

Comparative experiments were conducted on lung adenocarcinoma and breast cancer datasets to validate the method’s efficacy and generalizability. The results showed that the identified biomarkers were highly stage-specific, and the F1 scores for predicting cancer stages were significantly improved. For the lung adenocarcinoma dataset, the F1 score reached 97.68%, and for the breast cancer dataset, it achieved 96.87%. These results significantly surpassed those of three conventional methods in terms of F1 scores. Moreover, from the perspective of biological functions, the biomarkers were proved playing an important role in cancer stage-evolution.

**Conclusion:**

The proposed method demonstrated its effectiveness in identifying stage-related biomarkers. By using these biomarkers as features, accurate prediction of cancer stages was achieved. Furthermore, the method exhibited potential for biomarker identification in subtype analyses, offering novel perspectives for cancer prognosis.

## 1 Introduction

Cancer is a disease characterized by uncontrolled cell proliferation, posing a serious threat to human health. According to the World Health Organization, in 2020 alone, nearly 10 million people (about one-sixth of all deaths worldwide) died from cancer (Sung et al., 2021). Understanding cancer begins with an important dimension: its stages, which could describe the size and extent of tumor spread. Due to the high heterogeneity and complexity of cancer, it poses significant challenges for the identification of cancer stages (Burrell et al., 2013). Hence, investigating an intelligent model for the identification of stage-related biomarkers is very important. It helps in understanding the characteristics and changes during the development process of cancer. This research endeavor proves valuable in enhancing cancer treatment strategies and prognostic assessments.

As far as the biomarkers are concerned, encompass a range of molecules, cellular structures, or biological processes that can be objectively detected and quantified within or outside an organism (Moein et al., 2020). They play a crucial role in revealing an individual’s health status, physiological functions, pathological conditions, and biological responses to treatment. This makes them integral players in the development of precision medicine and personalized treatment strategies (Holland, 2016). Specifically, stage-related biomarkers provide crucial information about tumor progression, metastasis, and treatment response (Amin et al., 2010; Van der Kloet et al., 2012). By analyzing the expression patterns and changes of stage-related biomarkers, healthcare professionals and researchers can gain a better understanding of the cancer’s progression status, choose appropriate treatment strategies, and monitor treatment effectiveness.

However, molecular distinctions between different cancer stages are often subtle (Ye et al., 2020). For example, in early-stage cancer, molecular changes may be influenced by minor alterations in the activity of a few key genes or subtle modulation of signaling pathways. The boundaries between cancer stages, as defined clinically, are often indistinct at the molecular level. For instance, the molecular changes between stage I of a late-stage and stage II of an early-stage cancer could be very similar. Therefore, the identification of stage-related biomarkers at the molecular level has been a long-standing challenge.

Currently, two mainstream approaches primarily guide the identification of stage-related biomarkers. The first category is based on differential expression analysis. Deva Magendhra Rao et al. (2019) compared non-coding RNAs (lncRNAs) between invasive ductal carcinoma (IDC) breast cancer tissues and normal breast tissues. There were 375 differentially expressed lncRNAs identifying closely associated with the early-stage development of breast cancer. Shi et al. (2018) analyzed gene expression data from four stages of colorectal cancer, identifying stage-specific differentially expressed genes and exploring their shared biological functions. Wang et al. (2017) studied gene expression data in non-small cell lung cancer and found that differentially expressed genes at different stages significantly impacted biological functions and signaling pathways. However, these methods often overlook molecular interactions and typically validate their findings through functional or pathway enrichment analysis but few focus on the identification of stage-related biomarkers.

On the other hand, the second category, focuses on machine learning techeques. Patil and Bellary (2022) achieved good performance in stage identification of melanoma based on features from dermoscopic images and tumor thickness using machine learning. Ubaldi et al. (2021) performed a binary classification task to identify stage I and stage II non-small cell lung cancer using radiometric data and machine learning, achieving a high AUC value at 0.84. Jin et al. (2021) developed an interpretable machine learning model that could identify gene expression biomarkers for early-stage LUAD. However, these methods typically focus on building accurate prediction models similar to a “black box” with limited biological and clinical interpretability. Some researchers strive to construct interpretable machine learning models for identifying stage-related biomarkers, but this often leads to compromises in the predictive performance of the model to some extent for the samples are imbalanced, and there is minimal molecular-level difference between different stages. In summary, existent methods have weaker specificity in identifying stage-related molecular-level biomarkers.

In this paper, an efficient method was proposed to identify stage-related biomarkers through specific feature recognition on group specific networks (SFR-GSN), which could sensitively capture the differences between different stages and identify features that exhibit significant specificity between stages. Two mainly high-risk cancers, lung adenocarcinoma (LUAD) and breast carcinoma (BRCA), were used to evaluate the proposed method. Firstly, the clinical data, RNA-Seq data and protein-protein interactions (PPI) of LUAD and BRCA were first collected from public database. Then, based on the tumor samples and normal samples, the sample-specific networks (SSN) were constructed, which further intersected with PPI to construct the group-specific network (GSN). Through clinical data, GSNs were combined into one GSN corresponding to one cancer stage, which could address the weak specificity of existing biomarkers. Subsequently, a specific feature recognition (SFR) method based on these GSNs was proposed. SFR was designed in two-round, the first round was pre-screening by utilizing the random forest algorithm with Gini impurity quantifying the purity improvement. The second round was optimal subset screening of biomarkers by using the recursive feature elimination with cross-validation. Notably, during exploring the optimal results, the Cost-Benefit Ratio (CBR) was introduced as an important indicator for identifying the stage-related biomarkers. Eventually, comparative experiments among SFR-GSN and three state-of-the-art methods were conducted on LUAD and BRCA datasets to validate the effectiveness and generalization ability of the proposed method. The results showed that the identified biomarkers significantly improved F1 scores for predicting cancer stages. Also from the perspective of biological functions, the biomarkers were proved playing an important role in cancer stage-evolution.

## 2 Methods

### 2.1 Data collection

In the study, we focused on two kinds of cancer, lung adenocarcinoma (LUAD) and breast cancer (BRCA). On one hand, LUAD and BRCA are both cancer types associated with high levels of severity. LUAD is one of the most common subtypes of lung cancer, while BRCA is one of the most prevalent cancers among women. These two cancer types significantly impact patients’ quality of life and survival rates. On the other hand, since LUAD and BRCA are two common types of cancer with relatively high incidence rates worldwide, as a result, these cancer types have ample sample data available. The richness of data helps improve the accuracy and reliability of the models. Therefore, studying and analyzing datasets related to LUAD and BRCA can enhance our understanding of the disease mechanisms, risk factors, and treatment strategies, providing valuable insights for cancer diagnosis and treatment.

We separately collected the clinical data and RNA-Seq data of LUAD and BRCA from Xena Tomczak et al. (2015); Wang et al. (2022) and separated the RNA-Seq data into different pathological stages. Then, the counts per million (CPM) (Law et al., 2016) were applied to filter the low-expression genes, and genes with a value higher than 2 CPM in at least half of the samples were retained. Additionally, the protein-protein interactions were compiled from STRING (Szklarczyk et al., 2023). PPI was widely used in identifying biomolecules, including biomarkers, and driver genes in many studies. The RNA-Seq datasets used in the experiments is shown in Table 1.

**TABLE 1:** The number of samples of LUAD and BRCA in experiments.

Cancer types	Normal	Stage I	Stage II	Stage III	Stage IV	Sum
LUAD	59	273	122	83	26	563
BRCA	114	182	621	250	20	1,187

### 2.2 Construction of group specific networks

**FIGURE 1:**
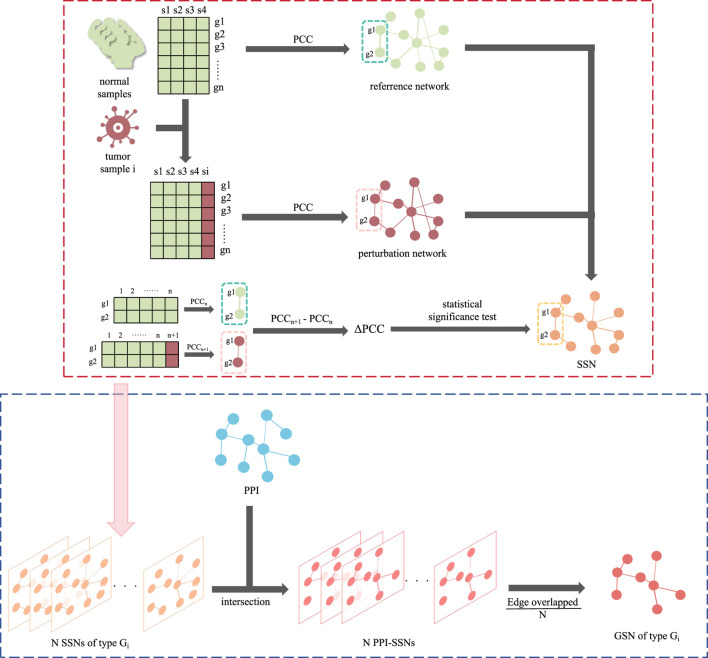
The flowchart of constructing GSN. The red dotted line section is the construction of SSN, while the blue dotted line section is the following part, using SSN and PPI to construct the GSN.

The group specific networks were constructed based on the two main kinds of networks: Sample-Specific Networks (SSN) and PPI networks. Proposed by Liu et al. (2016), SSN could assist in identifying driver genes from the perspective of the personalized network. GSN, combined SSN, and the existing PPI could increase the robustness of the interactions. The flow of the construction of GSN was summarized in Figure 1.

SSN was initially constructed based on RNA-Seq data. For all normal samples, a reference network was constructed by calculating the pairwise gene-gene Pearson correlation coefficients (*PCC*, represented in the reference network as *PCC*_*n*_). Meanwhile, for each disease sample, a perturbation network was generated by incorporating the normal sample set and reconstructing the network, resulting in *PCC*_*n*+1_. Subsequently, the differential network was obtained by subtracting the perturbation network from the reference network, and the difference was derived as Formula (1).

$$△PCC=PCC_{n+1}-PCC_{n}$$ (1)

Edges with a statistical *p*-*value* < 0.05 were considered significant and retained. In the constructed SSN, nodes represent genes, while the connections between nodes indicate significant differences in the correlation between the two genes in the disease sample compared to the normal sample set. This dissimilarity is quantified by △*PCC*.

Then, on the basis of the SSN, intersections were combined with the PPI. We retained the experimentally validated edges presented in PPI, with the edge weight calculated from SSN. Due to the samples could be divided into different pathological stages, the PPI-SSN for all samples was classified according to different stages (groups) of cancer. For instance, within one specific cancer group *G*_*i*_ responding to one GSN, consisting of *N* cancer samples, the *N* PPI-SSNs were integrated. Also, the edge weight was calculated by taking an average on the △*PCC* of same edge in *N* different samples. As for the edges not appearing in the samples, their △*PCC* was set to 0. Finally, the edge weight of GSN was derived as Formula (2).

$$w=\frac{\sum_{i=1}^{N}△PCC_{i}}{N}$$ (2)

Considering the generalization of GSN, a ten-fold cross-validation approach was employed during the experimental process. A GSN was constructed for each training fold, resulting in ten GSNs, and the edge weight from these ten GSNs was also averaged. Ultimately for every cancer group *G*_*i*_, only one corresponding GSN was constructed, which is stage-specific.

### 2.3 Specific feature recognition

Based on the constructed GSN, we aimed to identify the most representative and minimal set of features as biomarkers. These features in the selected set contain a high degree of complementary information, resembling a minimal control network. Feature recognition consists of two main parts: pre-screening and optimal subset screening of biomarkers.

#### 2.3.1 Pre-screening of biomarkers

The edge set of each GSN corresponding to each group is sorted in descending order based on the edge weights and subjected to pre-screening to obtain the top 50 edges. Among the top 50 edges, the features at both ends of these selected edges are obtained, and their union forms the candidate feature set. Then the candidate feature set is further filtered using the feature importance calculation algorithm embedded in random forest (Acharjee et al., 2020), narrowing it down to a new candidate feature set, which containing only the top 50 features based on their importance rankings. During the feature pre-screening, the Gini impurity was introduced to quantify the purity improvement achieved through branching. The Gini impurity, presented as *Gini*, could be derived as Formula (3).

$$Gini=1-\overset{n}{\underset{i=1}{\sum }}p_{i}^{2}$$ (3)

where *p*_*i*_ represents the relative frequency of the *i*-th class in the dataset, which is the probability of that class occurring in the dataset, and *n* is the total number of categories.

In random forest, the calculation of feature importance is based on the Gini impurity of each feature at each node in every tree. Specifically, for each feature, at each node of each tree, the algorithm splits the dataset into two subsets based on that feature. Then, the difference between the Gini impurity of the subsets after the split and the Gini impurity of the original node was calculated. Finally, by aggregating the feature importance scores from all nodes, the overall feature importance for each feature in the random forest was obtained. The built-in feature importance evaluation capability of the random forest makes it a powerful tool for understanding data and extracting key biomarkers in multi-class classification tasks. The whole pre-screening procession was described in Algorithm 1.

Algorithm 1. Pre-screening of biomarkers by random forest feature importance calculation.

**Require:** Random forest model *RF*, training data set *D*;
**Ensure:** A list of feature importances *importance*;
1: **for** each *tree* in *RF* **do**
2: **for** each *node* in *tree* **do**
3: **for** each *feature f* in *node* **do**
4: Split the dataset at *node* into two subsets *D*_*left*_ and *D*_*right*_ based on feature *f*;
5: Calculate the Gini impurity of the original node, denoted as Gini;
6: Calculate the Gini impurity of *D*_*left*_, denoted as *Gini*_*left*_;
7: Calculate the Gini impurity of *D*_*right*_, denoted as *Gini*_*right*_;
8: Calculate the gain in impurity after splitting on feature *f*:
9: $impurityGain=\frac{\left|D_{\mathrm{left}}\right|}{\left|D\right|}\times \left(Gini-Gini_{\mathrm{left}}\right)+\frac{\left|D_{\mathrm{right}}\right|}{\left|D\right|}\times \left(Gini-Gini_{\mathrm{right}}\right)$ ;
10: Update the importance of *feature f* based on the impurity gain:
11: *importance [f] ← importance [f] + impurityGain*;
12: **end for**
13: **end for**
14: **end for**
15: Sort the features based on the values in *importance* using a suitable sorting algorithm.

#### 2.3.2 Optimal subset screening of biomarkers

After the pre-screening, the top 50 candidate feature sets were further filtered by Recursive Feature Elimination with Cross-Validation (RFECV). The RFECV algorithm finds the optimal feature subset by iteratively removing features, involving model training and cross-validation for each reduced feature set. In each iteration, the algorithm removes the least important feature (the one contributing the least to the model’s performance improvement), retrains the model on the remaining feature set, and performs cross-validation. This process continues until a specific number of features is reached or further removal of features significantly degrades model performance.

Notably, to select the minimum number of features that achieve the best predictive performance, the Cost-Benefit Ratio (CBR) was introduced to assist in screening the optimal feature set (De Picker and Haarman, 2021). The CBR could be defined as Formula (4).

$$CBR=\frac{100\times PR}{INF\times UFC}$$ (4)

in this formula, the symbols represent the following:

• *PR*: Performance Gain, which refers to the improvement of the *F*1 score in the model.
• *INF*: Increased Number of Features.
• *UFC*: Unit Feature Cost.

Through CBR, we can quantitatively evaluate whether the performance improvement gained from adding specific features is worth the additional cost required. It is particularly important in situations where there is a need to balance decisions between performance improvement and cost.

During the model training, multiple thresholds (*thresh*) were set for the number of features and obtained their corresponding model performance evaluation metric, *F*1 score. Then, according to the CBR model, the optimal feature subset was screened in a recursive way. The optimal subset screening of biomarkers using RFECV was presented as Algorithm 2.

Algorithm 2 Recursive Feature Elimination with Cross-Validation (RFECV) algorithm.

**Require:** candidate feature set, threshold for the number of features *thresh*;
**Ensure:** feature set *S*, model performance evaluation metric *F*1 score;
1: Initialize the feature set *S* and set it as the candidate feature set;
2: Define the model performance evaluation metric *F*1 score;
3: Define the threshold for the number of features *thresh*;
4: **while** *S* is not ∅ **do**
5: Train the model using the feature set as the training set;
6: Introduce cross-validation to evaluate the model performance;
7: **if** the number of features = = *thresh* **then**
8: Save the current feature set as *S*;
9: Save the current model performance metric as *F*1 score;
10: *break*;
11: **end if**
12: Reove the least contributing features from *S*;
13: **end while**

## 3 Results

The experimental results were obtained using ten-fold cross-validation to ensure reliability. In each round, nine folds of the datasets were treated as a train set and the other one fold acted as a test set. The train set was used to construct the GSN and select the feature. The test set was utilized to evaluate the model performance. In addition, specific feature experiments and comparative analyses were conducted to validate the effectiveness of the model. Moreover, the proposed method was expanded to identify cancer subtypes related biomarkers as well.

### 3.1 Specific feature experiments

Specific feature experiments were conducted in the following two steps. Firstly, the important parameters were introduced including the CBR and number of features. Secondly, the stage-specific biomarkers in LUAD and BRCA datasets were identified. The effectiveness of the identified biomarker were performed through enrichment analysis.

#### 3.1.1 Setting of the important parameter

CBR was designed as a key parameter to assist in screening the optimal feature set, which is directly related to the number of features. The proposed methods were conducted on LUAD and BRCA datasets to determine a series of feature counts, and the F1 scores and CBRs were calculated through the experiments which was summarized in Table 2.

**TABLE 2:** F1 score and CBR for multi-class classification in stages of LUAD and BRCA at different feature quantity thresholds.

LUAD			BRCA		
Number of features	F1 score (%)	CBR	Number of features	F1 score(%)	CBR
1	48.7420	-	1	71.9124	-
2	86.2510	37.5090	2	93.4443	21.5318
3	91.2557	5.0047	3	95.3618	1.9174
4	92.5050	1.2492	4	96.2117	0.8499
5	93.3089	0.8039	5	97.2808	1.0691
6	96.1537	2.8448	6	97.4655	0.1846
7	96.8517	0.6979	7	98.2629	0.7974
8	96.9067	0.0550	8	**99.1047**	0.8417
9	97.4935	0.5868	9	98.3727	−0.7319
10	**97.8804**	0.3868	10	99.0001	0.6273

The bold values represent the best results among the column.

**FIGURE 2:**
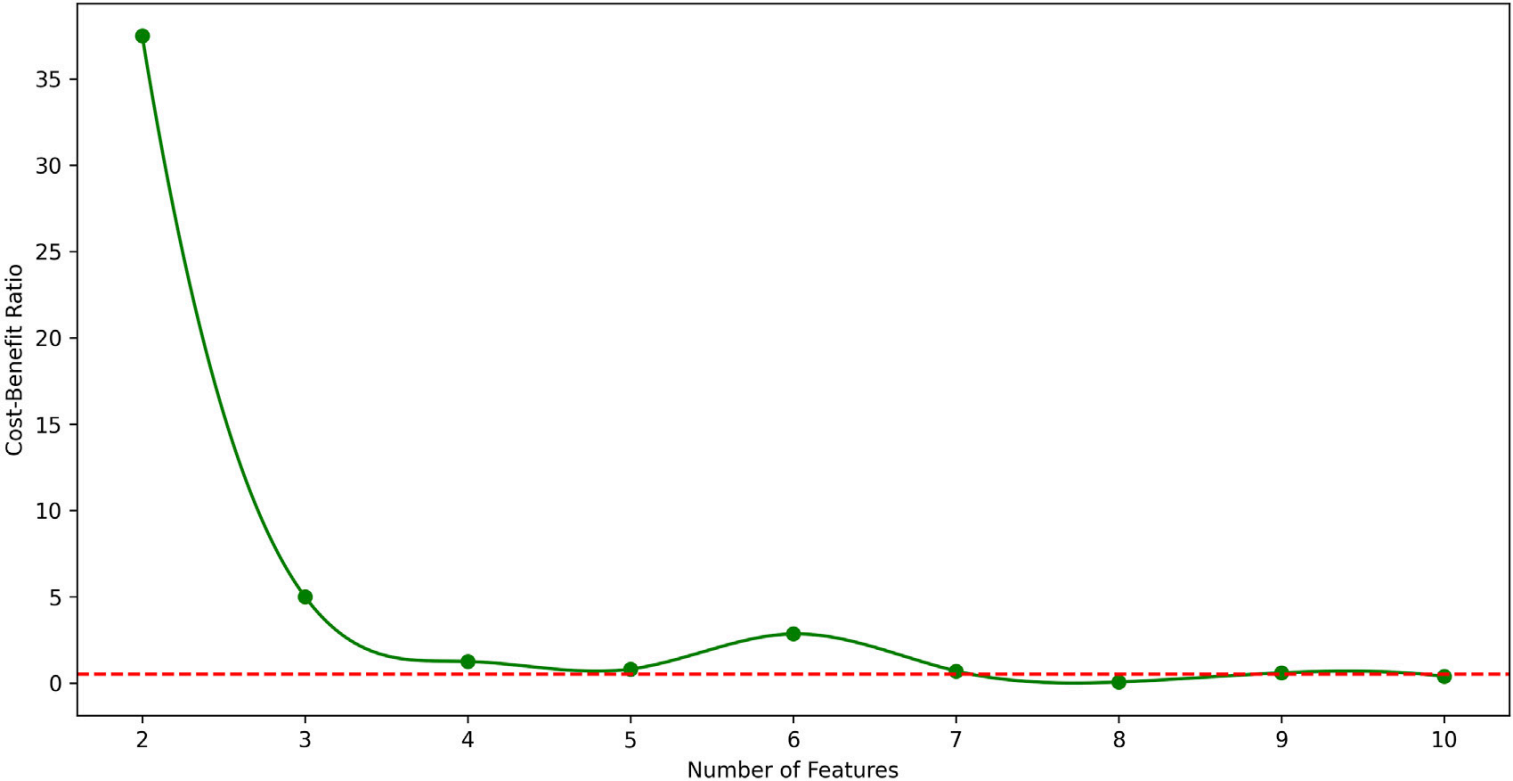
The relationship between *CBR* and number of features in LUAD datasets. The red dotted line represents *CBR* = 0.5 for parameter setting. From the figure, the best number of features in LUAD datasets is 7.

From the table, it is shown that in LUAD datasets, as the number of features increases, the F1 score generally improves, but the *CBR* shows non-monotonic variations. Therefore, to further illustrate the relationship between *CBR* and the number of features, their relationship in LUAD datasets was plotted in Figure 2. In the figure, the *CBR* values were compared with 0.5, as this threshold is often used as a balancing point. When the *CBR* is greater than 0.5, it indicates a profitable decision, while a *CBR* lower than 0.5 suggests a cost-effective decision.

Therefore, the *CBR* metric was utilized to determine the optimal number of features.

Starting with a small number of features and gradually increasing, the point was identified where the first *CBR* value fell below 0.5.

The CBR indicates the overall benefit of adding a new feature to the model. Therefore, the feature count just before this point was identified as the optimal number of features.

#### 3.1.2 Stage-specific biomarkers

Based on the parameter setting, features with CBR values greater than 0.5 were selected to maximize the F1 score. The obtained biomarkers were in the form of gene pairs or edges.

Compared with the node features, the edge biomarkers could better capture the interaction relationships between genes, aiding in understanding the structure and functionality of gene networks.

The edge features could reflect the interplay and coordinated regulation among genes, revealing more details about biological processes and disease development.

**FIGURE 3:**
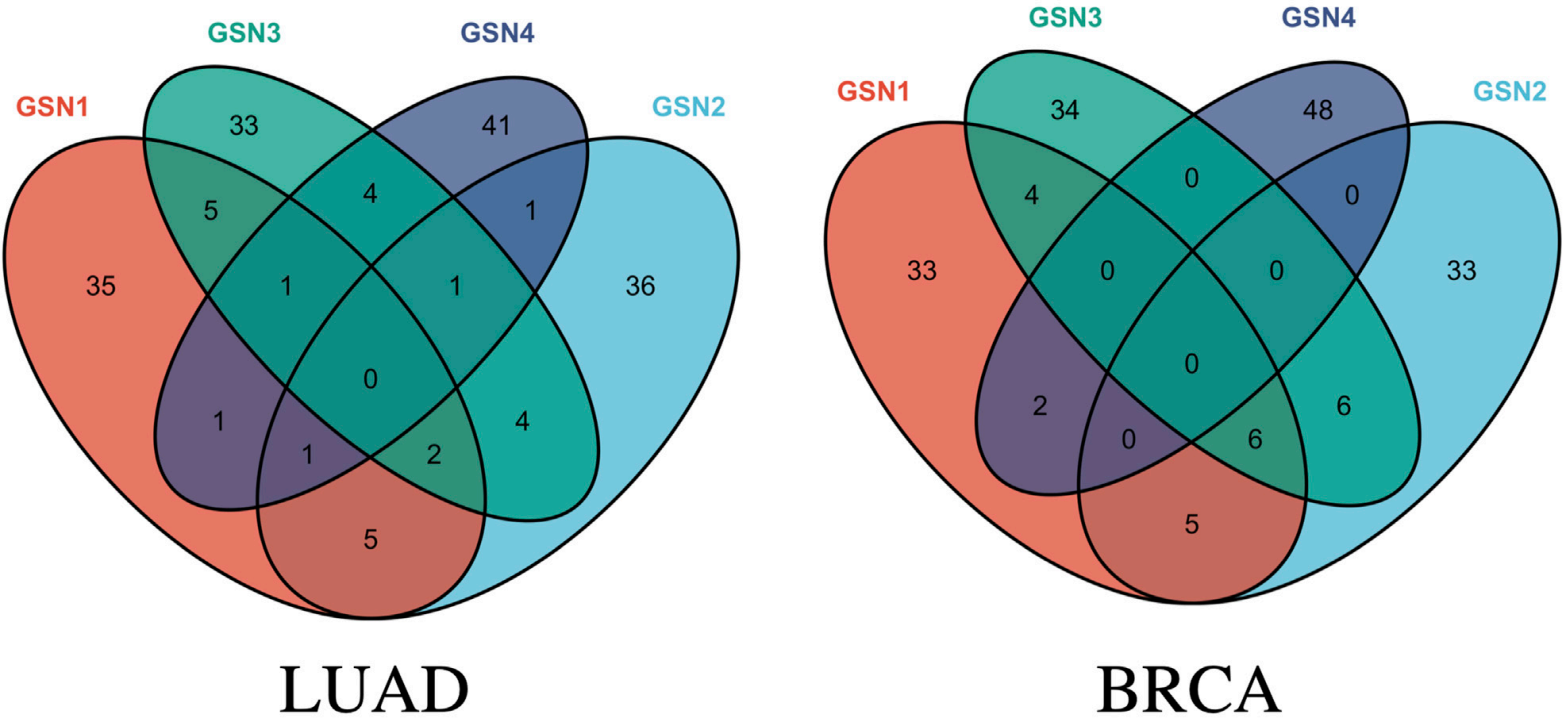
Venn graph of the obtained stage-related biomarkers for LUAD and BRCA.

As for the LUAD dataset, seven features were eventually identified that meet this criterion, achieving an impressive F1 score at 96.8517% and a CBR at 0.6979. These features include: (ABI2, ARPC1B), (CDK12, POLR2I), (FRS2, FRS3), (PABPC4, ZC3H14), (SNAP29, TSNARE1), (SEC24C, TRAPPC6B), and (CUL4A, RPA1). Similarly, for the BRCA dataset, five features were selected that yielded a remarkable F1 score at 97.2808% and a CBR at 1.0691. These features are: (EXOSC3, SKIV2L2), (BYSL, UTP14C), (EXOSC8, UTP14C), (PPP3CB, WDR82), and (CD59, SEC24C). The Venn graph of the obtained biomarkers is shown in Figure 3, which demonstrates the biomarkers were highly stage-specific.

### 3.2 Enrichment analysis

Moreover, the Kyoto Encyclopedia of Genes and Genomes (KEGG) Pathway analysis and Gene Ontology (GO) enrichment analysis were performed to validate the effectiveness of identified biomarkers.

**FIGURE 4:**
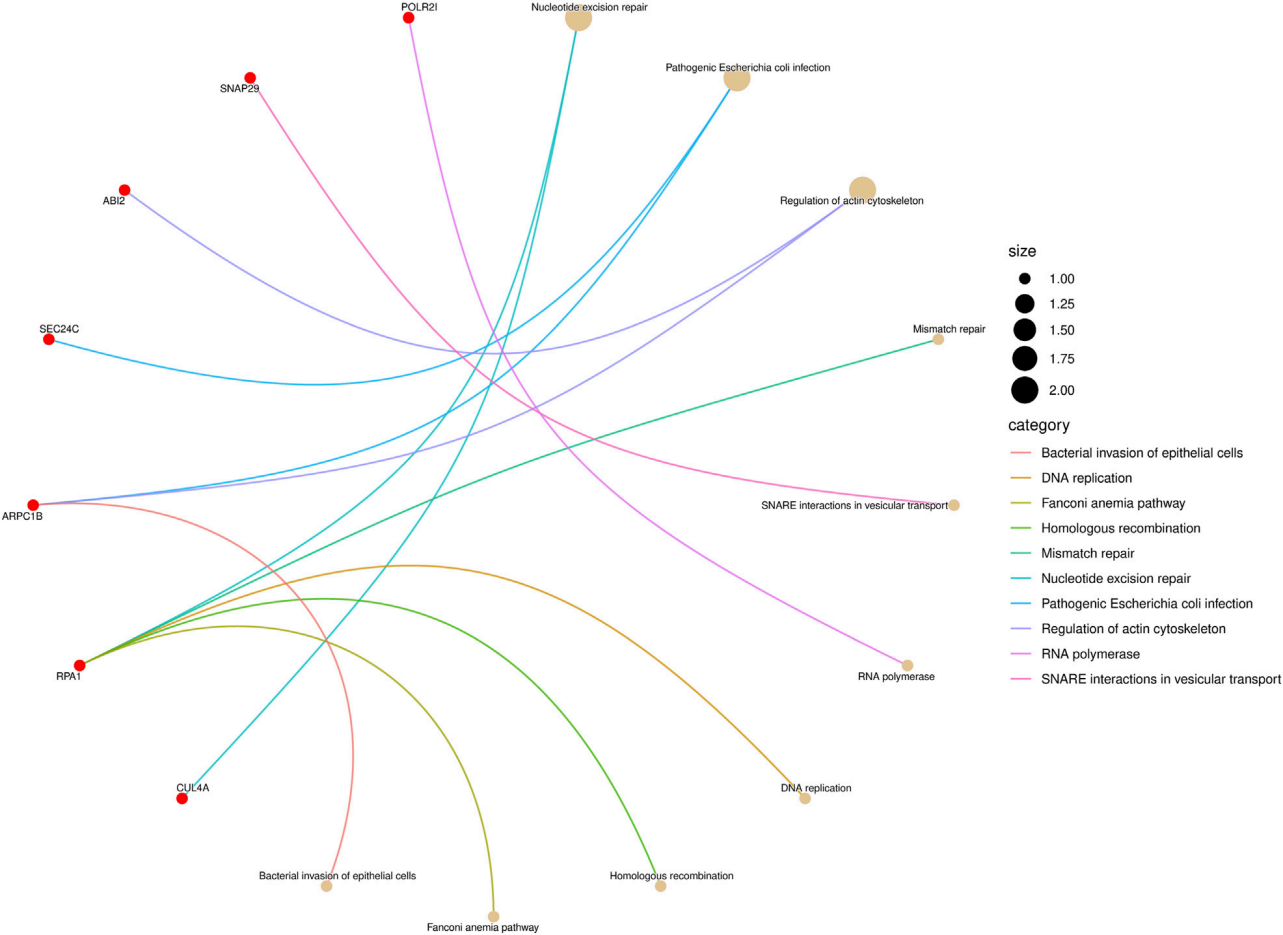
KEGG pathway enrichment result of LUAD stage-related biomarker.

KEGG pathway enrichment analysis is a frequently employed method in bioinformatics to interpret gene expression or protein expression data (Ogata et al., 1999). After performing a significance test on 14 genes in the biomarkers of LUAD stages, a total of seven genes were found to be enriched in 10 pathways. Among them, the gene RPA1 was found to be involved in five pathway processes, as shown in Figure 4. In the figure, the red dots represent genes, and the different colored curves represent different pathways. One end of the curve represents a gene, while the other end represents the hub of that pathway, and the size of the hub is proportional to the number of genes enriched in that pathway. As for stage-related biomarkers of BRCA, a total of three genes were found to be enriched in two pathways. Specifically, genes EXOSC8 and EXOSC3 were enriched in hsa03018: RNA degradation, while gene PPP3CB was enriched in hsa04370: VEGF signaling pathway. Due to the small number of genes, they were not visualized.

**FIGURE 5:**
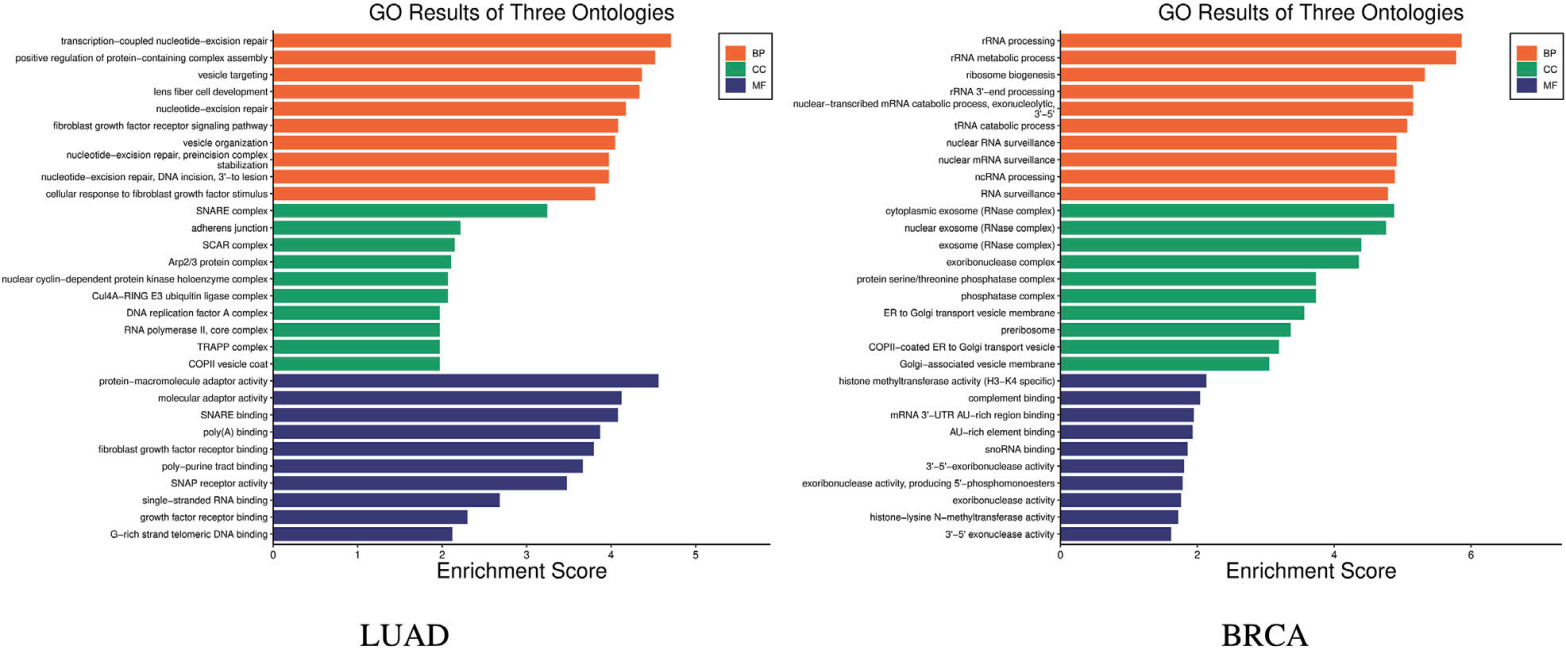
GO enrichment results of stage biomarkers for LUAD and BRCA.

GO (Gene Ontology) enrichment analysis was carried out to help understand the roles of a set of genes in biological processes Harris et al. (2004). GO enrichment analysis was carried out on the 14 genes in the stage-related biomarkers of LUAD, and the results are shown in Figure 5 LUAD, which indicates these 14 genes are involved in a total of 240 biological processes. In the figure, the *x*-axis represents the enrichment score, which indicates the degree of influence of the target genes on the corresponding GO term, while *y*-axis represents the different GO terms. The different colors represent the three main categories of GO. Each category includes only the top 10 terms based on their enrichment score. Similarly, GO enrichment analysis was performed on the nine genes in the stage-related biomarkers of BRCA, and the results are shown in Figure 5 BRCA. These nine genes were found to participate in a total of 205 biological processes.

The enrichment results demonstrate significant specificity of the features constructed using our proposed method across different stages within the two major cancer types, LUAD and BRCA. The evidence further validates the effectiveness of the proposed method.

### 3.3 Comparative experiments

Comparative experiments were conducted mainly in view of stage-related biomarker prediction. The proposed method was compared with the three conventional methods on biomarker identification: differentially expression genes (DEGs) Love et al. (2014), WGCNA Horvath (2011) and RelifF Robnik-Šikonja and Kononenko (2003). DEGs were mainly obtained using R package DESeq2 to conduct differential expression analysis, and the DEGs were treated as biomarkers. Based on differential expression data, WGCNA (Weighted Gene Co-expression Network Analysis) is a method used to construct co-expression networks from gene expression data, which is currently widely applied in the identification of biomarkers for complex diseases and drug targets. RelifF is a machine learning method on binary classification, which could identify the biomarkers.

Moreover, to ensure an equivalent comparison, the four methods were compared based on their best performance. Also, the features in all genes were performed as a control group. The F1 score was employed for evaluation since it is not influenced by the varying number of features across different methods. The results of the comparative experiments on LUAD datasets and BRCA datasets are shown in Table 3.

**TABLE 3:** The comparison of identification in stage-related biomarkers among SFR-GSN, three conventional methods, and all genes on LUAD and BRCA datasets.

	LUAD		BRCA	
Methods	Number of features	F1 score(%)	Number of features	F1 score(%)
All Genes	1,3326	38.90	1,3168	42.77
DEGs	225	42.42	318	42.51
WGCNA	151	40.35	396	43.89
Relife	100	42.29	100	43.49
SFR-GSN	7	**96.85**	5	**97.28**

The bold values represent the best results among the column.

From the table, it is shown that the proposed method significantly outperforms other methods in terms of F1 scores. Additionally, the proposed method provides fewer features than other methods, which indicates the proposed method could identify the biomarker more accurately.

### 3.4 SFR-GSN on cancer subtype-related biomarkers

Besides the evolutionary characteristics in different stages, cancer also exhibits various subtypes. As for LUAD, three types often occur in the evolution, which are Papillary Predominant (PP), Acinar Predominant (PI), and Trabecular (TRU). By studying subtype-related biomarkers, a better understanding of the differences in disease progression, treatment response, and prognosis among different subtypes could be obtained (Perou et al., 2000; Muller et al., 2022). Therefore, in order to enhance the generalization of our model, experiments on subtype-related data were conducted to identify the subtype-related biomarkers.

Firstly, the datasets were separated into the three subtypes and accordingly, three corresponding GSNs were constructed. Then, SFR was trained on the GSNs, features with CBR >0.5 were obtained, and the F1 score and CBR were shown in Table 4. Finally, five features were identified as subtype-related biomarkers of LUAD, these are (HDAC6, SIRT2), (AKT2, RICTOR), (DHX33, PINX1), (SNAP29, TSNARE1) and (ASPSCR1, VCPIP1). Similarly, the BRCA datasets were divided into five groups due to the five subtypes of BRCA. Eventually, the results were shown in Table 4, where six features were screened as subtype-related biomarkers, these are (SRC, USP8), (IRAK4, TOLLIP), (SRC, TRAF6), (F8, SEC24C), (CDK12, SUPT5H) and (CDC40, SF3B2).

**TABLE 4:** F1 score and CBR for multi-class classification in stages of LUAD and BRCA at different feature quantity thresholds.

LUAD			BRCA		
Number of features	F1 score(%)	CBR	Number of features	F1 score(%)	CBR
1	73.2800	-	1	51.2668	-
2	91.3155	18.0354	2	81.9457	30.6788
3	95.9758	4.1303	3	89.6415	7.6958
4	96.8973	0.5300	4	93.2176	3.5760
5	97.3499	0.9214	5	94.4989	1.2813
6	97.7847	0.4526	6	96.4546	1.9557
7	97.7847	0.4347	7	96.9095	0.4549
8	97.7847	0	8	97.3826	0.4731
9	97.7847	0	9	97.3919	0.0093
10	**98.2410**	0.4563	10	**97.7572**	0.3652

The bold values represent the best results among the column.

**FIGURE 6:**
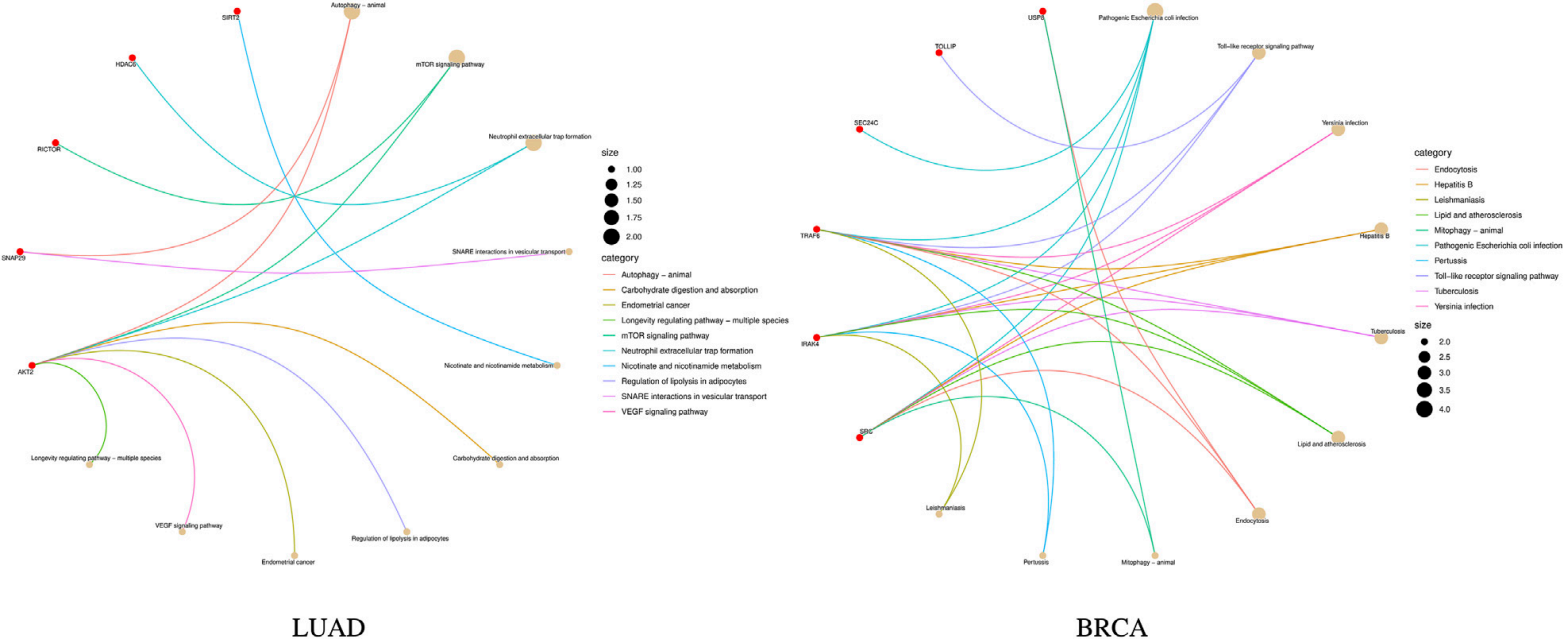
KEGG pathway enrichment results of subtype biomarkers for LUAD and BRCA.

**FIGURE 7:**
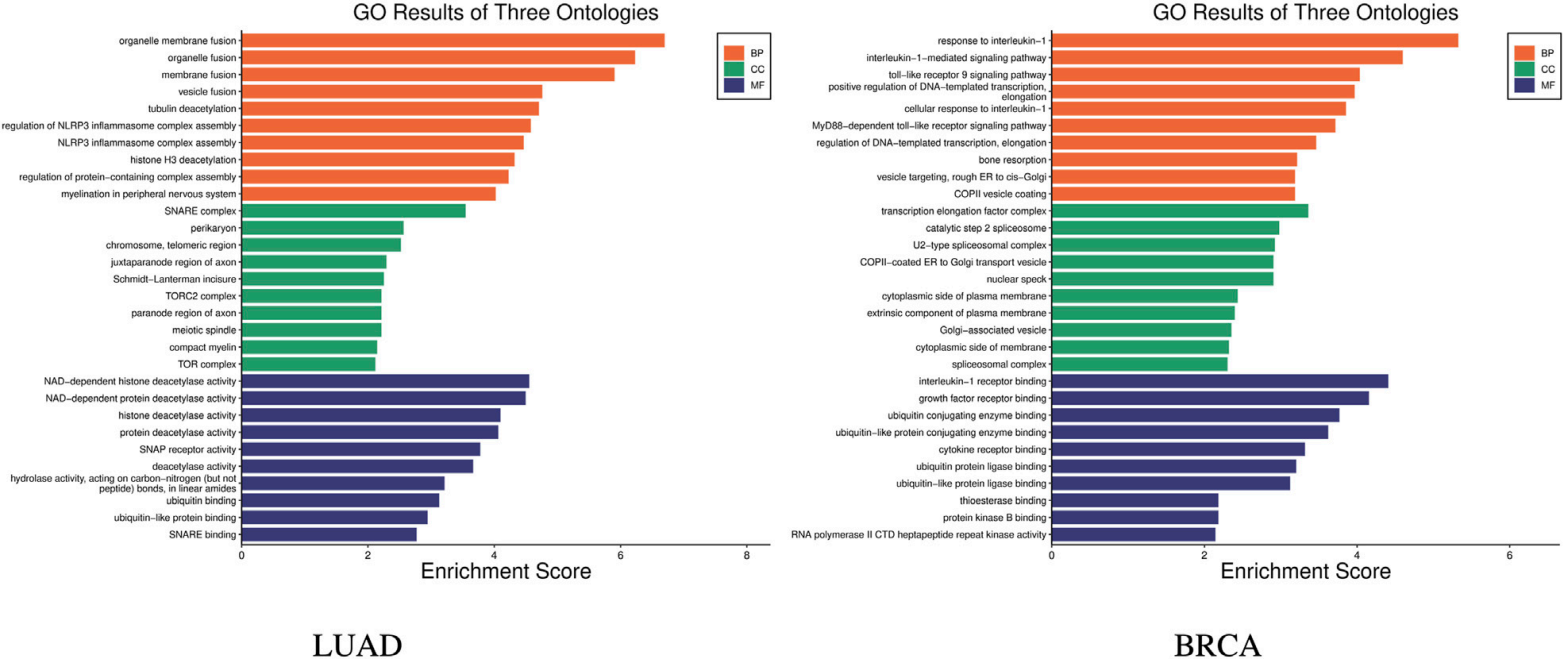
GO enrichment results of subtype biomarkers for LUAD and BRCA.

Further, the enrichment analysis was conducted on the identified features. In the subtype-related biomarkers of LUAD, five genes were enriched in 10 pathway pathways, with the gene AKT2 was found in eight pathway pathways. In that of BRCA, eight genes were enriched in 24 pathways, with the gene TRAF6 being enriched in 21 pathways and the gene IRAK4 was found in 20 pathways. KEGG pathway enrichment results are shown in Figure 6. Subsequently, the results of the GO enrichment analysis are shown in Figure 7. The 10 genes in the LUAD subtypes are involved in 360 biological processes, while 11 genes in the BRCA subtypes are involved in 407 biological processes.

After providing the results of SFR-GSN on the identification, the proposed method was also compared with three conventional methods and all genes. The results are shown in Table 5. SFR-GSN gains the best performance and the least features, which suggests SFR-GSN exhibits superior capability in identifying subtype-related biomarkers.

**TABLE 5:** The comparison of identification in subtype-related biomarkers among SFR-GSN, three conventional methods, and all genes on LUAD and BRCA datasets.

	LUAD		BRCA	
Methods	Number of features	F1 score(%)	Number of features	F1 score(%)
All Genes	1,3326	72.73	1,3168	85.9674
DEGs	2,478	82.38	3,922	87.77
WGCNA	426	78.00	632	86.32
Relife	100	81.16	100	83.45
SFR-GSN	5	**96.89**	6	**96.45**

The bold values represent the best results among the column.

## 4 Conclusion

In this work, a novel method called SFR-GSN has been proposed to identify the stage-related biomarkers, which gained remarkable results on LUAD and BRCA datasets. First, the clinical data, RNA-Seq data, and PPI were collected. Second, according to the pathological stage, the GSNs were constructed by combining the SSN and PPI. Third, based on GSNs, a two-round SFR was conducted, which firstly used random forest to pre-screen and later used RFECV to obtain the optimal feature sets. The CBR was introduced to assist in identifying stage-related biomarkers.

Finally, the results of the proposed method showed that the identified biomarkers were highly stage-specific and significantly improved the F1 scores for cancer stage prediction. For the lung adenocarcinoma dataset, the F1 score reached 97.68%, and for the breast cancer dataset, it achieved 96.87%. The results outperform the other conventional methods on both accuracy and F1 scores. Moreover, the enrichment analysis of biomarkers was conducted to validate the effectiveness of the proposed method in view of biological functions. The proposed method exhibits superior performance in identifying subtype-related biomarkers. The proposed method could be applied to other cancers to offer new insight into cancer treatment prognosis.

## Data Availability

The RNA-Seq data presented in the study are deposited in the UCSC Xena repository, accession number TCGA Lung Adenocarcinoma (LUAD) and TCGA Breast Cancer (BRCA), the url is https://tcga-xena-hub.s3.us-east-1.amazonaws.com/download/TCGA.BRCA.sampleMap%2FHiSeqV2.gz; the PPI data presented in the study are deposited in the STRING repository, accession number Homo sapiens, the url is https://stringdb-downloads.org/download/protein.physical.links.v12.0/9606.protein.physical.links.v12.0.txt.gz.
